# Fulminant hemangiopericytoma of the larynx – a case report and a review of the literature^☆^

**DOI:** 10.1016/j.bjorl.2017.04.007

**Published:** 2017-05-09

**Authors:** Lora Nikiforova, Nikolay Sapundzhiev, Penka Kolova, Georgi Boyadzhiev, Patrick Bradley

**Affiliations:** a Medical University Prof. Dr. P. Stoyanov, Department of Neurosurgery and Ear, Nose and Trought Diseases, Division of Otorhinolaryngology, Varna, Bulgaria; b Medical University Prof. Dr. P. Stoyanov, Department of Pathology, Varna, Bulgaria; c DRK Krankenhaus Altenkirchen-Hachenburg, Altenkirchen, Germany; d Queens Medical Centre, University Hospital, Combined Head and Neck Oncology Clinic, Nottingham, United Kingdom

## Introduction

Hemangiopericytoma (HPC) was first described in 1942 by Stout and Murray and represents a vascular tumor originating from Zimmerman's pericytes. These multi-potent cells encircle the endothelium of capillaries and venules. They play a role in the regulation of tissue microcirculation and are intrinsic for angiogenesis. Being stem cells in origin, they have the potential to differentiate into smooth muscle cells, macrophages, adipocytes, osteoblasts, or fibroblasts, but can also develop into malignant tumors (HPC's/solitary fibrous tumor, myopericytoma, glomus tumor).

HPC's may occur anyplace, but are predominantly located in the lower extremities, pelvis or retroperitoneum. They have a predilection for being located in the subcutaneous tissues and musculoskeletal system. Only 20–25% of all HPC's involve the head and neck region.^1^ Laryngeal involvement is a rarity, to the best of our knowledge, only 12 cases reported. Present is an additional case of an adult man whose laryngeal tumor rapidly progressed despite several local surgical interventions resulting in death.

## Case report

A 62-year-old Caucasian man presented with history of progressive dysphonia and dyspnea over 2 months. The patient had low to moderate exposure to standard risk factors, smoking and social alcohol, for Head and Neck Squamous Cell Carcinoma (HNSCC). Clinical examination revealed reddish polypoid mass, covered by apparently normal mucosa, occupying the left hemi-larynx. The mass at laryngoscopy appeared almost pedunculated with a narrow stalk based around the laryngeal ventricle – the lesion was excised *in toto* on the first occasion. Histology was reported initially as a poorly differentiated squamous cell carcinoma. The patient was informed of the diagnosis and the treatment options and discharged, as he needed time for consideration. Twenty-three days after the initial surgery the patient returned as an emergency with severe dyspnea. A huge mass was obstructing the laryngeal inlet, causing subtotal obstruction of the airway. This tumor re-growth necessitated an emergency tracheostomy. In the same setting a second “complete” resection was performed with microlaryngeal surgery. Ten days after this resection the patient was reviewed in another ENT-office, seeking a second opinion. Flexible endoscopy revealed a reddish mass on a narrow basis, measuring 8 mm × 8 mm on the left ventricular fold. The patient was referred to a tertiary ENT-clinic.

**Figure 1:**
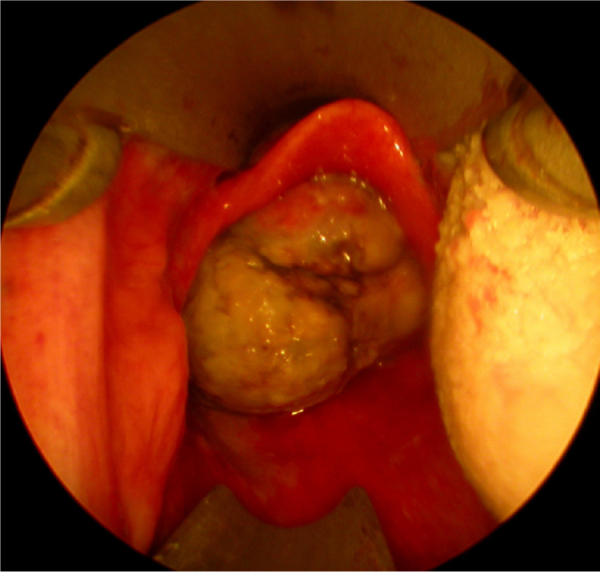
Endoscopic visualization of the laryngeal mass via Weerda diverticuloscope with the patient in supine position. The epiglottis appears completely normal above the intralaryngeal tumor mass.

**Figure 2:**
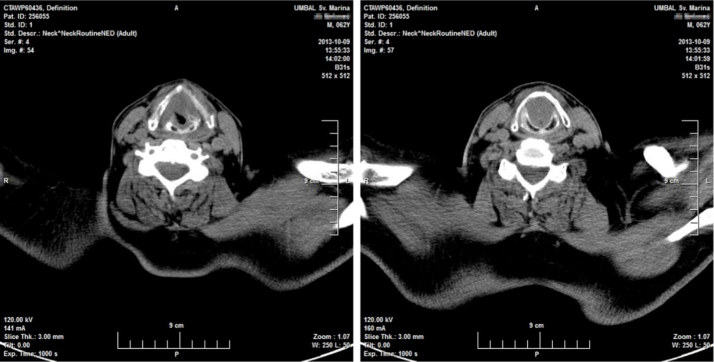
Axial computed tomography (CT) at the level of the thyroid (left) and cricoid (right) cartilages with complete lumen obstruction and no evidence cartilage invasion. The patient breathes through a tracheostomy.

Twenty days later the patient was admitted to our institution seeking further diagnostics and treatment. The tumor had regrown to complete laryngeal obstruction. The original pathology being reported as having atypical cellular appearance, there was a reluctance to proceed to performing a total laryngectomy, without additional biopsy and pathology review of the diagnosis. So the patient agreed and was subjected to a further endoscopic debulking to obtain tissue samples for repeated histology. The lesion was covered with smooth epithelium with large areas of ulceration and traces of bleeding (Fig. 1). This microlaryngeal R2 resection reduced the volume of the mass to about 15%. CT examination did not reveal any signs of regional or distant tumor spread, neither of invasions to the cartilage wireframe (Fig. 2). Meanwhile awaiting the final histology report the patient was discharged from hospital, with instruction on tracheostomy care.

**Figure 3:**
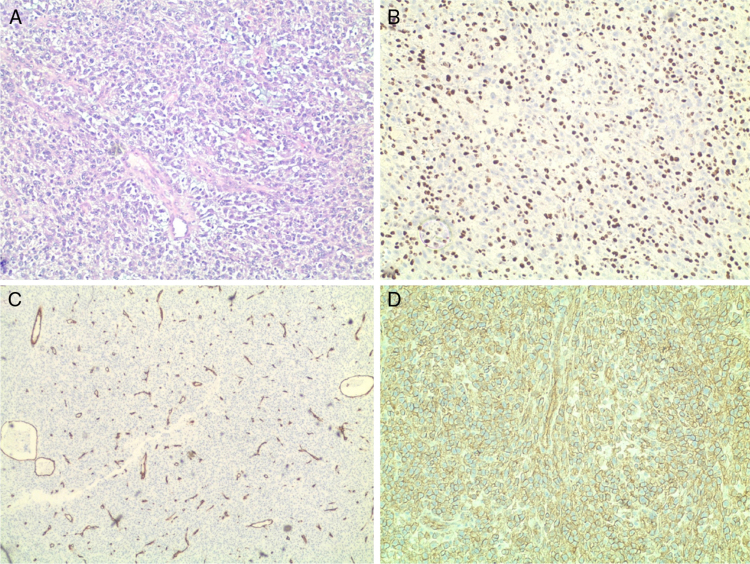
Histology images of hemangiopericytoma with different stains: (A) hematoxylin–eosin; (B) immunohistochemical stain for Ki-67; (C) immunohistochemical stain for CD34; (D) immunohistochemical stain for CD99.

Histology revealed the characteristic morphological features of hemangiopericytoma: abundance of thin-walled, ectatic “staghorn-like” branching capillaries and perivascular proliferation of monomorphic oval and spindle (elongated) tumor cells. Immunohistochemistry showed positive staining for Ki-67 (nuclear proliferation), CD99, CD34 (endothelial proliferation), S-100 protein, Vimentin, CD45 – all confirming that this is a malignant HPC. Ki-67 was expressed in the nuclei of 90% of the tumor cells, CD34+ were the endothelial cells (Fig. 3). The expression of S-100 protein was focal. Vimentin was expressed by the tumor cells in the perivascular regions. The tumor was negative for CKAE1/AE3, Chromogranin and Synaptophysin. This extensive pathology workup and the following tumor-board review took four weeks.

Attempts to make contact with the patient to explain the nature of his laryngeal disease, and to discuss further treatment options, failed as his relatives informed us, that the patient had died with evidence of bleeding from his tracheostomy.

## Discussion

With HPC estimated <1% of all head and neck tumors and the primary laryngeal location being a rarity, it is not surprising, that this clinical entity could be misdiagnosed. Primary laryngeal HPCs are a heterogeneous group with non-specific clinical features, so a clinical diagnosis itself is unlikely without histology confirmation. A review of the published literature, laryngeal HPC's presented macroscopically as polypoid or cyst-like masses beneath normal mucosa – with macroscopic features characteristic for benign laryngeal lesions.^1^, ^2^, ^3^, ^4^, ^5^ There is only one report, describing the laryngeal mass as probably malignant upon inspection.^6^ This reported case had been seen by 3 experienced laryngologists, all of them addressing the lesion as probably benign, and quite distinct from what macroscopically could be called the typical SCC of the larynx. HPC reported in proximal anatomic sites such as the trachea and nasal cavity are reported as presenting as a pedunculated polypoid mass.

The nonspecific macroscopic aspect of the lesion, the low-risk profile of the patient for HNSCC, and the untypically fast tumor re-growth after each microlaryngeal excision (retrospectively rather judged as extensive excisional biopsies) led to uncertainty about the initial histological diagnosis and necessity for second pathologic workup before decision for definitive treatment. The accurate histologic assessment is essential for the treatment of HPCs.^1^ HPC's possess distinctive microscopic characteristics – “staghorn-like” branching capillaries and perivascular spindle cells. Despite that HPC's may be confused with many other mesenchymal tumors (synovial sarcoma, solitary fibrous tumor, etc.), and possible confusion with other diseases specialized immunohistochemistry assays are considered essential to confirm the diagnosis. The term “hemangiopericytoma” appears ill-defined and inaccurate, as there are a lot of tumors with “HPC-like” features, which cannot be clearly defined as HPC. Now these tumors are included in the “solitary fibrous tumor (SFT) – hemangiopericytoma” group as HPC is now considered as the cellular form of SFT.

Once diagnosed and staged laryngeal HPC is best treated with radical laryngectomy. Published reports of laryngeal hemangiopericytomas reveal that patients subjected to total laryngectomy have a likely improved survival than those treated by partial laryngeal resection. Reviewing the 12 cases reported in literature, further information about the recurrence of the tumor is available for seven; 6 patients showed no evidence of disease during the follow-up (time range: 1–42 months after the surgery; median time: 21 months). Five patients underwent total or hemi-laryngectomy as first – or second-line treatment. One patient underwent pharyngotomy with total tumor excision.^2^, ^3^, ^4^ There is no reported evidence for additional treatments from radio- and/or chemotherapy. Only 2 patients of those 12 were treated with radiotherapy – one died after two radiation treatments, the other one (total dose of 6120 rads) was diagnosed with a “radiation-induced” squamous cell carcinoma 15 months after the operation. The clinical course and treatment outcome of the so far reported 12 cases of laryngeal HPC is summarized in Table 1.

**Table 1:** Summary of the 12 cases of laryngeal hemangiopericytoma reported so far.^1^, ^2^, ^3^, ^4^, ^5^, ^6^, ^8^, ^9^

Case	Author	Year	Treatment	Recurrence (+time)	Treatment	Follow-up
1	Stout	1956	–	–	–	–
2	Walike, Bailey^9^	1971	Due to the poor physical status, no surgery was conducted RT – two treatments	Multiple presentation of HPC's (lip, tongue, soft palate, epiglottis)	–	Died after 2 radiation treatments
3	Kuzniar	1971	–	–		
4	Taguchi et al.^2^	1974	Excision + postoperative RT (6120 rads)	No; “Radiation-induced” SCC 15 months postop	Total laryngectomy	27 months later: NED
5	Ferlito^6^	1978	None (poor condition)	–	–	Died from pulmonary abscess
6	Moncade, Demaldent^2^	1979	Excision	Yes, 8 months	Re-excision	–
7	Hertzanu et al.^3^	1982	Excision + preoperative embolization	–	–	–
8	Pesavento, Ferlito^4^	1982	Excision	Yes, 30 months later	Hemilaryngectomy	1 month later: NED
9	Schwartz, Donovan^5^	1987	Partial submucosal laryngectomy	No	No	3.5 years later: NED
10	Ey, Guastella^8^	1988	Lateral pharyngotomy Cricopharyngeal myotomy	No	No	6 months later: NED
11	Bradley et al.^1^	1989	Total laryngectomy Left hemi-thyroidectomy	No	No	40 months later: NED
12	Harirchian et al.^2^	2011	Preoperative embolization Partial pharyngectomy Partial supraglottic laryngectomy	No	No	10 months later: NED

NED, no evidence of disease; RT, radiotherapy.

However, there are some cases of HPC with other (unfortunately unspecified) localizations described in literature in which the radiotherapy itself has led to tumor disappearance.^7^ In other cases the tumor appeared to be radioresistant. Postoperative or palliative radiotherapy is often used for HPC treatment, unlike chemotherapy, which role in tumor growth control is not yet fully investigated.^7^

A meta-analysis of 116 patients with primary HNHPC in 2014 defined surgical removal as optimal first-line treatment. Non-surgical treatment modalities were given as one of the main independent adverse prognostic factors for survival together with deep-seated tumor location and size >5 cm in diameter.

The data about the growth rate of HPC are scarce. In the first report Stout and Murray (1942) defined the growth of HPC as “locally persistent, aggressive, infiltrative”, rarely with metastases. HPC's with most common localizations (retroperitoneum, pelvis) are considered slow-growing. Intra-osseus HPC's have extremely slow growth rate and the asymptomatic period may reach 20 years. In the meninges HPC is usually a fast-growing tumor with high local recurrence. Head and neck located HPC may significantly vary in their rate of growth, but the majority of the described cases define it as a relatively slow-growing. Head and neck HPC's include: orbital (“slow growing”), sinonasal (“slow growing”), of the oral cavity (“slow-growing”) and the parotid gland (“slow-growing”).

Only 5 of the 12 reports of HPC's of the larynx contain information on the rate of tumor growth – HPC is described as a slow-growing tumor with long asymptomatic period. However, this is not always a statement proven by the presented cases, but rather information gained from the cited references, included in the discussion part of the articles.^3^, ^5^, ^6^, ^8^, ^9^ If concerning the airway in general HPC's of the nasal cavity and trachea are characterized by a symptomatic course of 3 months to 2 years before diagnosis. The tracheal HPC re-grew to a symptomatic tumor after 5 months. Against this background, in our case the laryngeal HPC presented as a tumor mass with a very rapidly growth/re-growth, with local progression marked by airway compromise and bleeding. Within a period of 54 days the tumor had two complete macroscopic excisions and had re-grown on two occasions with impending acute life-threatening airway obstruction. Over the last time period of observation, 20 days between office endoscopy and last MLS, the lesion size increased from about 8 mm × 8 mm to 27 mm × 24 mm, causing complete laryngeal obstruction (Fig. 1).

## Conclusion

HPC of the larynx is an extremely rare neoplasm with unpredictable biological behavior, debatable treatment and is a diagnosis of exclusion. The criteria to make a diagnosis of a HPC malignancy remain controversial, including histological characteristics and its clinical course. In most sites HPC appears to be slow-growing, with a benign appearance, and it is only with histological expertise can a conclusive diagnosis be made. This reported case of laryngeal HPC was characterized with a fulminant course, dominated by the high local growth/re-growth rate, without evidence of regional or distant spread.

## Conflicts of interest

The authors declare no conflicts of interest.
